# Photoluminescence and Stability of 2D Ruddlesden–Popper Halide Perovskites

**DOI:** 10.3390/molecules30132716

**Published:** 2025-06-24

**Authors:** Zhilin Ren, Zhengtian Yuan, Aleksandr A. Sergeev, Ivor Lončarić, Muhammad Umair Ali, Atta Ur Rehman, Kam Sing Wong, Yanling He, Juraj Ovčar, Jasminka Popović, Aleksandra B. Djurišić

**Affiliations:** 1 Department of Physics, The University of Hong Kong, Pokfulam Road, Hong Kong; 2 Department of Physics, William Mong Institute of Nano Science and Technology, The Hong Kong University of Science and Technology, Clearwater Bay, Hong Kong; 3 Ruđer Bošković Institute, Bijenička 54, 10000 Zagreb, Croatia; 4 Material Characterization and Preparation Facility, The Hong Kong University of Science and Technology (Guangzhou), No. 1 Duxue Road, Dongchong Town, Nansha District, Guangzhou 511453, China

**Keywords:** lead halide perovskite, photoluminescence, stability

## Abstract

Two-dimensional lead halide perovskites are of significant interest for a variety of practical applications. However, the relationships between their composition and properties are not fully clear. Here we investigated photoluminescence from 2D Ruddlesden–Popper perovskites with different bulky spacer cations. Significant differences in their optical properties and stability are observed, and perovskites with benzylammonium (BZA) and phenethylammonium (PEA) were selected for more detailed investigation of the observed stability differences due to their similar structure. We find that PEA_2_PbI_4_ exhibits more narrow emission and increased stability compared to BZA_2_PbI_4_. In addition, PEA_2_PbI_4_ exhibits self-healing of defects evident from PL enhancement, which is absent for BZA_2_PbI_4_. The observed differences between perovskites with BZA and PEA spacer cations can be attributed to differences in the formation of spacer cation vacancies.

## 1. Introduction

Due to their exceptional properties, 2D lead halide perovskites have been attracting increasing interest in recent years for a variety of practical applications [1,2,3,4,5,6,7,8], including solar cells, light emitting diodes (LEDs), photodetectors, X-ray detectors, etc. [1,2]. These materials consist of a lead cation, halide anion X, and a bulky organic cation (A), which is too large to fit into cavities between corner-sharing lead halide octahedra and form a 3D perovskite, resulting in a 2D perovskite with the formula A_2_PbX_4_ for Ruddlesden–Popper (RP) 2D materials [1]. Quasi-2D RP perovskite films, with the formula A_2_C_n−1_Pb_n_X_3n+1_, consist of *n* layers of 3D perovskites with small cations (C) separated by bilayers of bulky A cations. The case of *n* = 1 (no small cation), corresponds to 2D perovskites, while *n* = ∞ corresponds to 3D perovskites. The properties of RP perovskite materials are significantly affected by the choice of organic cations, [1] and yet their structure–property relationships remain largely unexplored due to a large number of organic cations consisting of an ammonium head and a variety of organic tails that can form 2D RP perovskites [1]. While efforts have been made to compare properties of perovskites with different cations for both lead-based [3,8,9,10,11,12,13,14,15,16,17,18,19,20,21,22,23,24,25,26,27,28] and tin-based [29] perovskites, further work is still needed to improve our understanding of the relationships between spacer cation structure and resulting perovskite properties, including perovskite stability. While 2D perovskites are commonly assumed to lead to improved stability compared to 3D perovskites [1,2,4], the stability of 2D perovskites with different spacer cations can exhibit significant variations [8]. In addition, despite the claims of the high stability of 2D perovskites, bulky cations can diffuse from a 2D to 3D perovskite [5], and ion migration, in general, is present in 2D perovskites [7]. Nevertheless, the use of 2D perovskites typically results in a measurable increase in device stability [4]. Two-dimensional perovskites are commonly used in solar cells in 3D/2D absorber layers, which typically have higher efficiency compared to quasi-2D based devices, while quasi-2D perovskites are of more interest in light-emitting diode applications due to their stronger light emission compared to 2D perovskites [6]. As quasi-2D perovskites typically contain multiple phases [1] and exhibit phase transformation during degradation [8], 2D perovskites that do not contain small cations are more suitable for investigations of the relationships between spacer cation and perovskite optical properties and stability.

The optical properties of different 2D RP lead bromide perovskites have been recently reviewed [3], and the stability of different RP perovskites under illumination in dry air for both bromide and iodide perovskites has been investigated [8]. However, comprehensive investigations of the relationships between photoluminescence (PL) and stability have been scarce, and studies comparing different cations typically investigate smaller numbers of cations. Nevertheless, it is known that the choice of the spacer cation can have a significant effect on 2D perovskite properties. For example, spacer cations can significantly affect the crystallization [14], phase composition [14], and stability [19] of quasi-2D films, as well as the halide migration [8,9], photoluminescence (peak position, intensity, and linewidth) [10,12,15,16,20,23,26], trap states [13], mechanical properties [22,27], deformation in the distances and bond angles of PbI_6_ octahedra [17,27], band structure/band alignment [17,24], spacer cation dynamics [18], and stability (thermal [11,21], ambient [20,27], and light [8,21,28]) of 2D perovskites. As spacer cations clearly have a significant effect on the properties of the corresponding 2D perovskites, it is of significant interest to examine 2D perovskites with different spacer cations. Therefore, here we examined the photoluminescence and stability of 2D perovskite films (bromide and iodide) for eight different spacer cations. We observed that 2D iodide perovskites exhibit larger variation in their PL spectra, and selected two spacer cations, namely benzylammonium (BEA) and phenethylammonium (PEA), for a more detailed investigation due to the similarity of their chemical structures.

## 2. Results and Discussion

**Figure 1:**
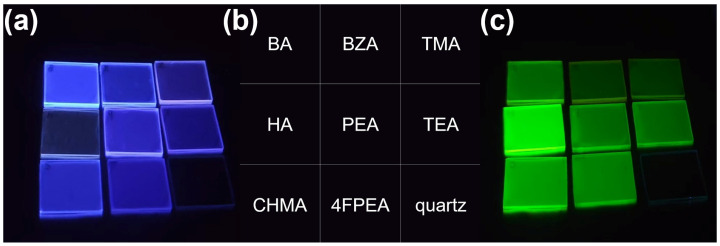
(**a**) Photos of A_2_PbBr_4_ 2D RP perovskites under UV lamp; (**b**) corresponding A cations for different sample positions; (**c**) photos of A_2_PbI_4_ 2D RP perovskites under UV lamp. BA denotes butylammonium, BZA denotes benzylammonium, TMA denotes 2-thiophenemethylammonium, HA denotes hexylammonium, PEA denotes phenethylammonium, TEA denotes 2-thiopheneethylammonium, CHMA denotes cyclohexanemethyl ammonium, and 4FPEA denotes 4-fluoro-phenethylammonium.

Figure 1 shows the representative photos of different bromide and iodide perovskites, while the corresponding representative normalized PL spectra are shown in Figure S1.

The absorption spectra, XRD patterns, and FTIR spectra of as-prepared samples and samples illuminated in ambient air for 2 h for A_2_PbBr_4_ and A_2_PbI_4_ are shown in Figures S2–S4 and Figures S5–S7, respectively. The films with different spacer cations and the same halide anions have similar thicknesses, as summarized in Table S1 and observed from the scanning electron microscopy (SEM) images of sample cross-sections shown in Figures S8 and S9.

We can observe that the brightness trends vary for the same spacer cations and different halide anions, which could possibly occur due to differences in crystal structure parameters. For example, it is known that the intensity of broadband emissions in halide perovskites is correlated with bond distortions as this type of emission arises from self-trapped excitons [3]. These emissions are typically very broad, with linewidths on the order of hundreds of nanometers, different from near-band-edge narrow emissions, with linewidths on the order or tens of nanometers. For near-band-edge narrow emissions, the rigidity of the cation affects charge carrier relaxation and recombination, ultimately resulting in differences in emission intensity and linewidth [3,23]. All the samples investigated here exhibit near-band edge emission. Among the investigated samples, the most pronounced difference occurs for HA cations, where the lowest emission is obtained from HA_2_PbBr_4_ and the highest from HA_2_PbI_4_. However, as the HA-based perovskite films were prepared using different solvents to ensure good film quality since smooth, uniform films could not be prepared from pure N,N-dimethylformamide solutions, the emission intensity could be affected not only by the film composition but also by the preparation conditions. From the normalized PL spectra, we can observe that the peak positions are dependent on the spacer cations. In the case of bromides, we observe similar widths of emission peaks for all spacer cations. In contrast, for iodides, we observe not only variations in the peak positions but also variations in the peak shapes for different spacer cations. For the CHMA cation, we observe two emission peaks in iodide perovskites, while for all other spacer cations, we observe single-emission peaks with different linewidths. Dual-emission peaks were previously reported in different 2D perovskites [10,11,13], and attributed to bulk and surface states [13,30,31], or free and self-trapped excitons (STEs) [10]. Since we have not observed dual-emission peaks for some materials previously claimed to exhibit dual-emission peaks (BZA_2_PbI_4_ and PEA_2_PbI_4_) [10], it is possible that these emissions originate from defect states or phase impurities dependent on the fabrication process. It should also be noted that despite the reported assignment of lower energy peaks in the PL spectra of BZA_2_PbI_4_ and PEA_2_PbI_4_ to STEs [10], the Stokes shift and linewidth of these peaks are smaller than typically associated with STE emissions.

**Figure 2:**
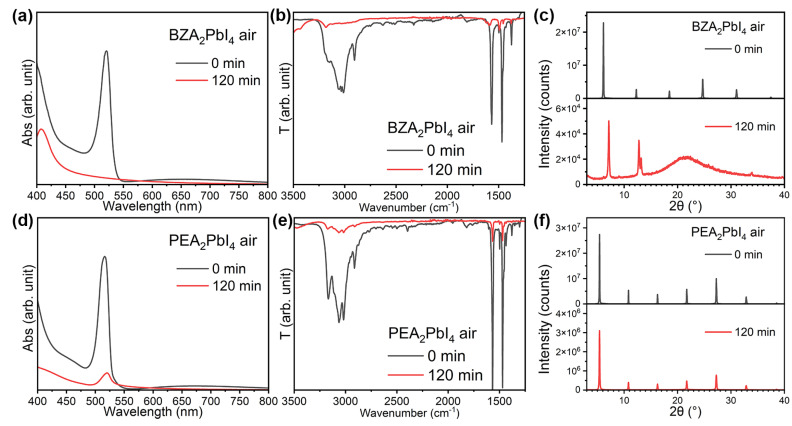
(**a**) Absorption spectra, (**b**) FTIR spectra, and (**c**) XRD patterns of BZA_2_PbI_4_ before (0 min) and after (120 min) 1 Sun illumination in ambient air; (**d**) absorption spectra, (**e**) FTIR spectra, and (**f**) XRD patterns of PEA_2_PbI_4_ before (0 min) and after (120 min) 1 Sun illumination in ambient air.

We can also observe that perovskite stability in an ambient air atmosphere (~60% relative humidity) under illumination (Figures S2–S7) follows similar trends to stability under illumination in dry air [8]. BZA_2_PbI_4_ exhibits worse stability compared to PEA_2_PbI_4_, as shown in Figure 2. In all cases, degradation under illumination is associated with the loss of organic cations based on FTIR spectra (Figure 2, Figures S4 and S7), in agreement with our previous work [8]. From the XRD patterns, however, we can observe that in some cases ambient illumination of iodides results in the appearance of peaks corresponding to phases different from the 2D RP phase, and for those samples, we generally observe either broader PL spectrums or the appearance of an additional peak, even in the cases where secondary phases cannot be clearly resolved in the XRD pattern before illumination.

To investigate the effects of spacer cations on light emission and stability under illumination, we need to select cations with similar chemical structure and perovskite film quality, as multiple factors can affect the optical properties of perovskites with different spacer cations. Thus, here we selected BZA_2_PbI_4_ and PEA_2_PbI_4_ as representative examples of perovskites with wide and narrow emission spectra, respectively, as these two materials only differ in the number of carbon atoms connecting the ammonium group to the phenyl ring. We have investigated the effects of sample fabrication procedures, and we found that vacuum drying and annealing significantly affected the stability of BZA_2_PbI_4_, while the two procedures resulted in similar properties for PEA_2_PbI_4_ films, as shown in Figure S10. As annealed films also exhibit higher PL intensity for both BZA_2_PbI_4_ and PEA_2_PbI_4_, indicating better initial sample quality, as shown in Figure S11, we have selected annealed films for a more detailed investigation of light emission and stability under illumination, and therefore all the data in the following section correspond to annealed films.

From the comparison of PL spectra of BZA- and PEA-based perovskite films (Figure S11), we can also observe that for all fabrication conditions, a larger full width at half maximum (FWHM) is obtained for BZA_2_PbI_4_ compared to PEA_2_PbI_4_. Significant differences in the shape and width of the emission peaks of 2D perovskites have been reported in the literature. These materials typically exhibit high exciton binding energies, exceeding 200 meV [6]. Thus, it would be expected that sharp emission peaks would be observed in PL spectra. Indeed, exfoliated crystals exhibit narrow emission spectra, while thin films exhibit broader emission lines, with PL linewidths of ~80 meV for exfoliated crystals and ~240 meV for thin films with butylammonium spacer cations [6]. Thin films, in general, exhibit broader emissions compared to single crystals, due to inhomogeneous broadening arising from a broad distribution of crystallite thicknesses within the film [32]. It was also previously reported that BA_2_PbI_4_ exhibited larger FWHM of the PL peak at room temperature compared to PEA_2_PbI_4_ and also exhibited a larger increase in FWHM and a non-radiative recombination rate increase with rising temperature, which was attributed to higher electron–phonon coupling for BA [23]. In addition, the broad emission in BA_2_PbI_4_ was also attributed to defect states [33]. In general, line broadening in 2D perovskites at temperatures > 100 K can be attributed to temperature-independent inhomogeneity caused by crystal imperfections Γ_0_ and temperature-dependent homogeneous broadening due to exciton–longitudinal optical phonon scattering [12,16].

However, it is also worthwhile noting that broad emissions in this work, as well as in the literature, have been observed in 2D perovskites that are not stable under illumination (BA, BZA) [8,28]. To examine the relationship between emission properties and stability, we have also investigated the stability of BZA_2_PbI_4_ and PEA_2_PbI_4_ in nitrogen under UV illumination, as PL measurements are conducted in nitrogen under short-wavelength excitation, different from common stability investigations commonly conducted in ambient air under 1 Sun illumination.

**Figure 3:**
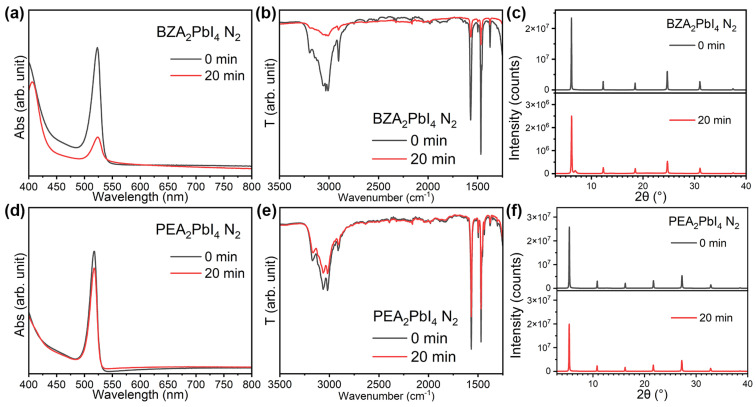
(**a**) Absorption spectra, (**b**) FTIR spectra, and (**c**) XRD patterns of BZA_2_PbI_4_ before and after 20 min UV illumination (365 nm) in nitrogen; (**d**) absorption spectra, (**e**) FTIR spectra, and (**f**) XRD patterns of PEA_2_PbI_4_ before and after 20 min UV illumination (365 nm) in nitrogen.

From the comparison of stability in air (Figure 2) and nitrogen (Figure 3), we can observe improved stability for PEA_2_PbI_4_ compared to ambient air, while for BZA_2_PbI_4_, significant degradation is observed even under a nitrogen atmosphere. A noticeable loss of organic cations in BZA_2_PbI_4_ is evident in both N_2_ and ambient air, as shown in Figure 2b and Figure 3b. This indicates that the loss of organic cations plays a significant role in the perovskite degradation and that this process is independent on the presence of oxygen and moisture. This is in agreement with the observation of the loss of BA^+^ cations under the illumination of quasi-2D perovskites both in air and in N_2_ [34]. In addition, it was reported that the loss of BA^+^ cations in 2D and quasi-2D perovskites under illumination occurs faster than the loss of MA^+^ cations, based on FTIR measurements and PL mapping [35]. Lower photostability compared to MAPbI_3_ was also reported for PEA_2_PbI_4_ [36], in contrast to expectations of improved stability in devices incorporating 2D perovskites. In addition, the BA_2_PbI_4_ 2D perovskite was found to be less stable compared to both the 3D MAPbI_3_ and quasi-2D BA_2_MA_n−1_Pb_n_I_3n+1_ perovskites (for *n* = 2 and *n* = 3) [37]. Furthermore, the expulsion of both Br^−^ and I^−^ was reported for BA_2_PbBr_2_I_2_ films under illumination, in contrast with 3D perovskites, where only expulsion of I^−^ was observed [38]. The observed instability under illumination for 2D perovskites raises concerns about their long-term stability [7,38], illustrating the need for a better understanding of the photostability of this class of materials.

**Figure 4:**
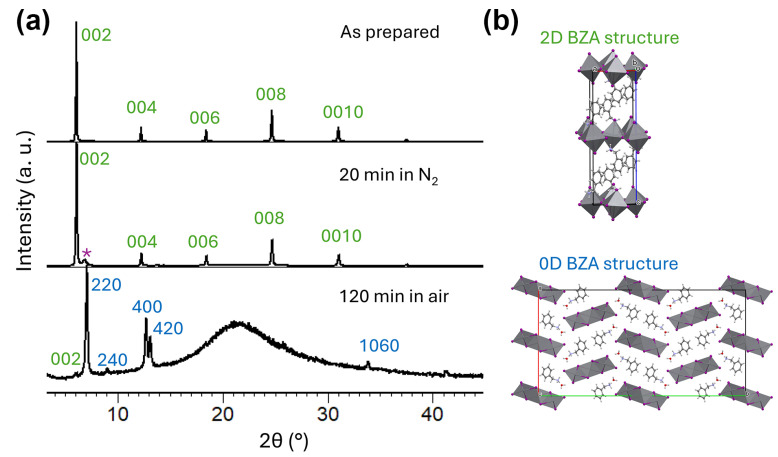
(**a**) XRD patterns of as-prepared and illuminated BZA_2_PbI_4_ films; (**b**) schematic diagrams of 2D and 0D structures formed with BZA spacer cations. Purple asterisk denotes unidentified phase. Lead polyhedra are shown in gray, iodide atoms are represented by purple balls, and C, N, and H atoms are given by gray, blue, and white balls, respectively.

Thus, our results on low photostability for BZA_2_PbI_4_ are consistent with previous literature reports on the stability of 2D perovskites under illumination [8,37,38,39,40]. The lower stability of BZA_2_PbI_4_ compared to PEA_2_PbI_4_ was also predicted by DFT calculations and attributed to a shorter carbon chain and/or a lower number of carbon atoms for BZA (one for BZA, two for PEA) [27]. It should be noted, however, that any stability comparisons between organic cations can only be made if the cations are forming the same type of structure (2D RP perovskite). Lower stability of BZA-based compared to PEA-based perovskites applies not only for Pb-based perovskites but also for Sn-based perovskites, where lower stability was attributed to longer Sn-I and N-H···I bonds and weaker interplanar interactions for BZA [29]. It can also be observed that in addition to the loss of organic cations evident from the reduced intensity of N-H vibration peaks, BZA_2_PbI_4_ also exhibits structural transformation from a 2D to 0D structure, as illustrated in Figure 4. The XRD patterns given in Figure 4a show that as-prepared BZA_2_PBI_4_ exhibits a strong preferred orientation with periodicity along *c* = 28.781(3) Å, similar to that reported by Kanatzidis et al. [39]. We can observe that exposure to 1 Sun illumination for 20 min in nitrogen leads to a decrease in X-ray intensity, indicating degradation of the 2D structure accompanied by the appearance of a small unidentified XRD peak at ~6.87°2*θ*. After exposure to 1 Sun illumination for 2 h in ambient air, the 2D BZA structure transforms into the hydrated 0D perovskitoid structure BZA_2_Pb_3_I_8_∙2H_2_O, as shown in Figure 4b [40]. The perovskitoid structure consists of inorganic Pb-I slabs separated by BZA cations and water molecules that are mutually connected via hydrogen bonding. On the other hand, though in Figure 2d some decrease in intensity can be seen, the PEA-based sample retains its 2D structural assembly (with unit cell parameter *c* = 17.67(1) Å), which agrees with previously published reports on its structure [41] after 1 Sun illumination for 2 h in ambient air.

**Figure 5:**
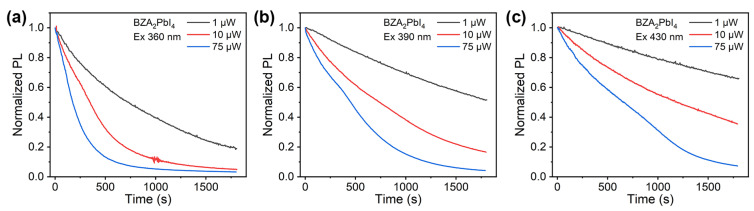
Normalized PL spectra of BZA_2_PbI_4_ films in N_2_ as a function of time for different excitation powers and excitation wavelengths of (**a**) 365 nm, (**b**) 390 nm, and (**c**) 430 nm.

**Figure 6:**
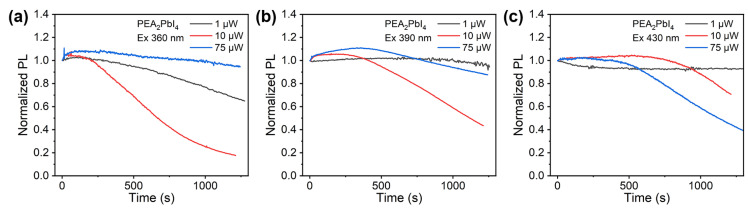
Normalized PL spectra of PEA_2_PbI_4_ films in N_2_ as a function of time for different excitation powers and excitation wavelengths of (**a**) 360 nm, (**b**) 390 nm, and (**c**) 430 nm.

In addition to a larger FWHM, BZA_2_PbI_4_ also exhibits a slower decay of time-resolved photoluminescence compared to PEA_2_PbI_4_, as shown in Figure S12. The relevant parameters for the characterization of light emission properties are summarized in Table S2. While the TRPL decay traces are complex to interpret and reported PL decay times can vary significantly from one lab to another due to experimental protocol differences for both fabrication and characterization, comparing PL decays of samples prepared in the same lab can still provide useful information about the samples [42]. For both samples, TRPL traces do not show significant dependence on the excitation wavelength. However, there is a dramatic difference in the intensity of PL emission as a function of time, as shown in Figure 5 and Figure 6 for BZA_2_PbI_4_ and PEA_2_PbI_4_, respectively. In the case of BZA_2_PbI_4_, for all excitation wavelengths and excitation powers, the intensity decays over time. The decay is faster for shorter excitation wavelengths and higher power. In contrast, PEA_2_PbI_4_ exhibits more complex behavior with clear photo-brightening behavior (PL intensity first increases and then decreases with time) for different combinations of excitation wavelengths and powers. More intriguingly, this behavior is more pronounced at higher excitation power. For 430 nm excitation, low power (1 µW) does not lead to self-healing, but it also does not lead to a significant decay of intensity after the initial drop. Photo-brightening and self-healing were reported to occur in both quasi-2D and 3D perovskite materials [43,44], but no differences in self-healing behavior were observed for different spacer cations. Interestingly, while brightening is observed for quasi-2D BA_2_MA_n−1_Pb_n_I_3n+1_, no brightening was observed for 2D *n* = 1 BA_2_PbI_4_ [44]. Thus, brightening of PL was attributed to the presence of MA^+^ [44], and it was proposed to occur by defect-passivating coordination of Pb to the N lone pair in MA^+^, while the incomplete self-healing of BA_2_PbI_4_ was attributed to the irreversible loss of BA [44]. From the similarity in the behavior of BZA_2_PbI_4_ to the behavior previously reported for BA_2_PbI_4_, namely broad emissions attributed to defect states [33], the loss of spacer cations as a cause of instability [35], and the lack of photo-brightening [44], the spacer cation loss could explain the observed behavior.

We have previously established that the main reason for the observed stability differences between RP and DJ perovskites is the fact that cation vacancies readily form under illumination in DJ perovskites, resulting in the loss of cations, as evidenced by FTIR spectra, and the outgassing of cation degradation products [28]. From the results in this work, the spacer cation obviously has a significant effect on the formation of cation vacancies, since BZA is readily lost even in a N_2_ environment, as shown in Figure 3b. We can also observe that in a nitrogen environment, there is no change in FTIR peak shapes or their relative intensity to each other, different from longer illumination in air, which indicates that phase transformations are less likely for shorter illumination times or in the absence of moisture, similar to observations in quasi-2D perovskites, which exhibited differences in phase changes under illumination in ambient and dry air [28].

Thus, to further investigate differences in organic vacancy formation between PEA_2_PbI_4_ and BZA_2_PbI_4_, we follow a methodology described in detail in our previous investigation [28]. For our initial structures, we construct 2 × 2 × 1 supercells from the experimentally obtained crystal structures of PEA_2_PbI_4_ and BZA_2_PbI_4_ [45,46]. Following structural optimization (as described in the Computational Method Section), we model deprotonated structures by moving one hydrogen atom from the ammonium head of the PEA/BZA organic spacer to an axial position between two iodine atoms. The lower bound on the energy required to deprotonate a spacer is then estimated as

$$E_{f}^{dep}=E^{dep}-E^{0},$$

where $E^{dep}$ and $E^{0}$ are the energies of the optimized deprotonated and initial structures, respectively.

Then, we remove the deprotonated organic spacer from the optimized deprotonated model structure. The estimated lower bound on the organic vacancy formation energy is

$$E_{f}^{vac}=E^{vac}-{(E}^{dep}+E^{mol}),$$

where $E^{vac}$ is the energy of the optimized structure with the removed deprotonated organic spacer, and $E^{mol}$ is the energy of the optimized deprotonated organic spacer in vacuum.

The calculated values of $E_{f}^{dep}$ and $E_{f}^{vac}$ for PEA_2_PbI_4_ and BZA_2_PbI_4_ are shown in Figure S13. $E_{f}^{dep}$ for PEA_2_PbI_4_ is larger by approximately 0.12 eV compared to BZA_2_PbI_4_. This could possibly be attributed to the interaction between the positively charged ammonium group and the aromatic π-electronic system, which is permitted in PEA_2_PbI_4_ due to the folded molecular conformation, while it is absent in BZA_2_PbI_4_ due to the shorter carbon chain [47].

Furthermore, $E_{f}^{vac}$ is also found to be larger in PEA_2_PbI_4_ by approximately 0.15 eV, in agreement with the experimentally observed stability trend. To fundamentally resolve the physical mechanism of PEA_2_PbI_4_ stabilization versus BZA_2_PbI_4_ destabilization, a detailed investigation, employing a more precise approach (e.g., density functional theory), is warranted to correlate the stacking configurations of the organic spacers with the strength of intermolecular and organic–inorganic interactions.

Taking into account experimental results demonstrating clear differences in the loss of organic cations between BZA- and PEA-based perovskites, which is consistent with theoretical predictions on cation vacancy formation, we propose that this difference likely explains the observed photoluminescence behavior. In BZA_2_PbI_4_, BZA^+^ cations are rapidly lost, resulting in an irreversible loss of luminescence, similar to the previously reported case of BA_2_PbI_4_. In contrast, in the case of PEA_2_PbI_4_, cation loss still occurs, albeit at a significantly slower rate. PEA^+^ cation migration would thus enable defect passivation by coordination of Pb to the N lone pair of PEA^+^, similar to the proposed mechanism of photo-brightening for MA^+^ [44]. With prolonged illumination and the eventual loss of PEA^+^, the luminescence intensity would then decrease. Complex dependences between excitation wavelength, excitation power, and the PL intensity variation over time are likely a result of the interplay between existing non-radiative defects and ion migration under illumination, with the exact mechanism requiring further study.

## 3. Materials and Methods

### 3.1. Experimental Techniques

Ultraviolet absorption (UV-vis) spectra were measured with an Agilent Cary 60 UV-Vis spectrometer (Agilent Technologies, Santa Clara, CA, USA). The in situ absorption spectrum was measured on a home-build setup of a monochromator equipped with 1350 ln/mm grating and a CMOS photodetector. The spectrum was calculated from the Beer–Lambert law, A = log_10_(I/I_0_), where A is absorbance, I_0_ is the light intensity before crossing the perovskite film, and I is the light intensity after crossing the perovskite film. In situ absorption measurements were performed under 100 mW/cm^2^ simulated solar illumination (Abet Technologies Sun 2000 Solar Simulator, Milford, CT, USA) in ambient conditions (relative humidity RH~60%). X-ray diffraction (XRD) patterns were measured by a Rigaku MiniFlex X-ray Diffractometer with CuKα radiation (Rigaku Corporation, Tokyo, Japan). The scanning step was 0.01° with a 10°/min scanning speed. Perovskite cross-section SEM images were obtained using a Hitachi Regulus 8230 FE-SEM (Hitachi HighTech, Tokyo, Japan) with 5.0 kV accelerating voltage.

FTIR spectra of thin films were obtained using a Spectrum Two ATR-FTIR spectrometer (PerkinElmer, Norwalk, CT, USA). Corresponding blank substrates were used as background. Perovskite films were measured before and after aging under 100 mW/cm^2^ simulated solar illumination in ambient conditions (RH around 60%) or 100 mW/cm^2^ UV (365 nm) in nitrogen atmosphere.

Steady-state and time-resolved PL measurements were carried out by exciting samples with 100 fs laser pulses generated by frequency doubling the output of a mode-locked Ti:sapphire oscillator. The excitation wavelengths were 360, 390, and 430 nm, and the repetition rate for laser pulses was 2000 kHz. The beam was attenuated by a variable ND wheel, and the power before the sample was measured with a laser power meter (Newport 1936c, Irvine, CA, USA). The laser was focused onto the sample (spot size 100 µm), and the PL signal was collected into a monochromator (Acton Spectrapro 275, Teledyne Acton Optics, Acton, MA, USA) by a pair of achromatic lenses, equipped with an EMCCD (Andor Newton, Belfast, UK) and a time-correlated single-photon counter (Becker and Hickl, Berlin, Germany). To collect spectra, the PL signal was dispersed by a 150 ln/mm grating onto the EMCCD. PL kinetics were measured by continuously collecting PL spectra (0.05–0.1 s integration time, depending on the excitation power to maintain the spectrometer in a linear mode) for 10 accumulations over 30 min. The PL intensity of each time point was integrated from 480 to 650 nm, and the intensity was normalized based on the first integrated intensity data. PL decay traces were collected using a 1200 ln/mm grating tuned at the PL central wavelength, which dispersed the signal onto a photon counter. PL measurements were carried out in dry nitrogen at 23 °C.

### 3.2. Synthesis

*Materials*: N,N-dimethylformamide (DMF, anhydrous, 99.9%), dimethyl sulfoxide (DMSO, anhydrous, 99.9%), and γ-butyrolactone (GBL) were purchased from Thermo Fisher Scientific Chemicals (Waltham, MA, USA). Lead(II) bromide (PbBr_2_, ≥98%) and lead(II) iodide (PbI_2_, ≥98%) were purchased from Tokyo Chemical Industry Co., Ltd. (TCI, Tokyo, Japan). Benzylammonium bromide/iodide (BZABr/I, >99%), phenethylammonium bromide/iodide (PEABr/I, >99%), *n*-butylammonium bromide/iodide (*n*-BABr/I, >99%), *n*-hexylammonium bromide/iodide (*n*-HABr/I, >99%), 2-thiophenemethylammonium bromide/iodide (TMABr/I, >99%), 2-thiopheneethylammonium bromide/iodide (TEABr/I, >99%), 4-fluoro-phenethylammonium bromide/iodide (4FPEABr/I, >99%), and Cyclohexane methylammonium bromide/iodide (CHMABr/I, >99%) were purchased from Greatcell Solar, Queanbeyan, Australia. Acetone (99.5%) and IPA (99.5%) for substrate cleaning were purchased from Anaqua (Wilmington, DE, USA).

*Perovskite Film Fabrication*: Perovskite thin films were fabricated on quartz substrates (20 mm × 20 mm), which were consequently cleaned in detergent, deionized water, acetone, and IPA for 30, 30, 30, and 10 min, respectively, in an ultrasonic bath and blown dry with nitrogen. Finally, the surface was treated with oxygen plasma for 1 min. BZAI was mixed with lead iodide PbI_2_ in a 2:1 mole ratio and dissolved in DMF solvent to obtain a BZA_2_PbI_4_ precursor solution with a 0.2 M lead concentration. Two-dimensional perovskite BZA_2_PbI_4_ thin film was prepared by spin-coating the precursor solution on a cleaned quartz substrate at 4000 rpm for 30 s, followed by vacuum drying at −1.0 bar pressure for 1 h to remove the solvent residue or by annealing at 100 °C for 10 min. Other 2D perovskite films were prepared in the same way with corresponding monoammonium halide salt (AX, with A for PEA, BA, HA etc., and X for Br or I) instead of BZAI; for example, PEAI was used to obtain the precursor solution of PEA_2_PbI_4_. For all 2D perovskite films prepared in this work, DMF was used as the only solvent for precursor solution, except for HA_2_PbBr_4_ (DMF:DMSO = 9:1) and HA_2_PbI_4_ (DMF:GBL = 85:15), to achieve films with good quality. The spin-coating parameters and post-treatment were the same for all 2D perovskite films.

*Computational Method*: All calculations were performed using an ensemble of MACE equivariant message passing neural networks [48,49], trained specifically to model low-dimensional hybrid organic–inorganic perovskites [50]. All structural optimizations were performed using the Fréchet cell filter, as implemented in the Atomic Simulation Environment software package [51], by relaxing the atomic positions and unit cell parameters until the force on each atom was less than 1 meV/Å.

## 4. Conclusions

We investigated the optical properties and ambient stability of A_2_PbBr_4_ and A_2_PbI_4_ perovskites for different A spacer cations. We found that spacer cations had a significant effect on stability under illumination and on light emission. A_2_PbI_4_ perovskites exhibited worse stability compared to bromides, as well as more diverse line shapes and line widths of the emission spectra. Thus, BZA_2_PbI_4_ and PEA_2_PbI_4_ were selected for a more detailed investigation of optical properties and stability. The inferior stability of BZA_2_PbI_4_ was attributed to the unimpeded loss of organic cations, even in a nitrogen environment. BZA_2_PbI_4_ also exhibited a larger FWHM of the PL spectrum and a slower TRPL decay compared to PEA_2_PbI_4_, as well as the absence of photo-brightening behavior for all excitation wavelengths and excitation powers. In contrast, PEA_2_PbI_4_ exhibited photo-brightening behavior, which can likely be attributed to spacer cation diffusion within the film leading to defect passivation. As PEA^+^ loss occurs with prolonged illumination, photo-brightening is typically followed by a decrease in intensity.

## Data Availability

Data are contained within the article and Supplementary Materials.

## Supplementary Materials

The following supporting information can be downloaded at: https://www.mdpi.com/article/10.3390/molecules30132716/s1, Figure S1: PL spectra of different iodide and bromide perovskites. Figures S2–S7: Absorption spectra, XRD patterns, and FTIR spectra of A_2_PbBr_4_ and A_2_PbI_4_ for different organic cations A. Figures S8 and S9: SEM cross-section images of different perovskite films. Figure S10: Absorption spectra of annealed and vacuum-dried BZA_2_PbI_4_ and PEA_2_PbI_4_ for different times. Figure S11: PL spectra of annealed and vacuum-dried BZA_2_PbI_4_ and PEA_2_PbI_4_. Figure S12: TRPL traces measured for different excitation wavelengths for annealed BZA_2_PbI_4_ and PEA_2_PbI_4_. Figure S13: Estimated lower bounds on the deprotonation and organic spacer vacancy formation energies for BZA_2_PbI_4_ and PEA_2_PbI_4_. Table S1: Film thickness values for different perovskite films. Table S2: Light emission parameters for BZA_2_PbI_4_ and PEA_2_PbI_4_.
