# Supplementary material for: Photoluminescence and Stability of 2D Ruddlesden–Popper Halide Perovskites
Source: Molecules. 2025 Jun 24;30(13):2716. doi: 10.3390/molecules30132716 (PMC12250890; doi:10.3390/molecules30132716)
Supplement: Supplementary file 1 [file molecules-30-02716-s001.zip › molecules-3680138-supplementary.pdf]

SUPPLEMENTARY INFORMATION

Photoluminescence and stability of 2D Ruddlesden-Popper halide perovskites

Zhilin Ren,[1#] Zhengtian Yuan,[1#] Aleksandr A. Sergeev,[2] Ivor Lončarić,[3] Muhammad Umair Ali,[1] Atta Ur Rehman,[1] Kam Sing Wong,[2] Yanling He,[4] Juraj Ovčar,[3,*] Jasminka Popović[3,*] and Aleksandra B. Djurišić[1,3*]

[1] Department of Physics, The University of Hong Kong, Pokfulam Road, Hong Kong

[2] Department of Physics and William Mong Institute of Nano Science and Technology, The Hong Kong University of Science and Technology, Clearwater Bay, Hong Kong

[3]Ruđer Bošković Institute, Bijenička 54, Zagreb, Croatia

[4]Material characterization and preparation facility, The Hong Kong University of Science and Technology (Guangzhou), No.1 Duxue Road, Dongchong Town, Nansha District, Guangzhou, Guangdong Province, China

#These authors contributed equally.

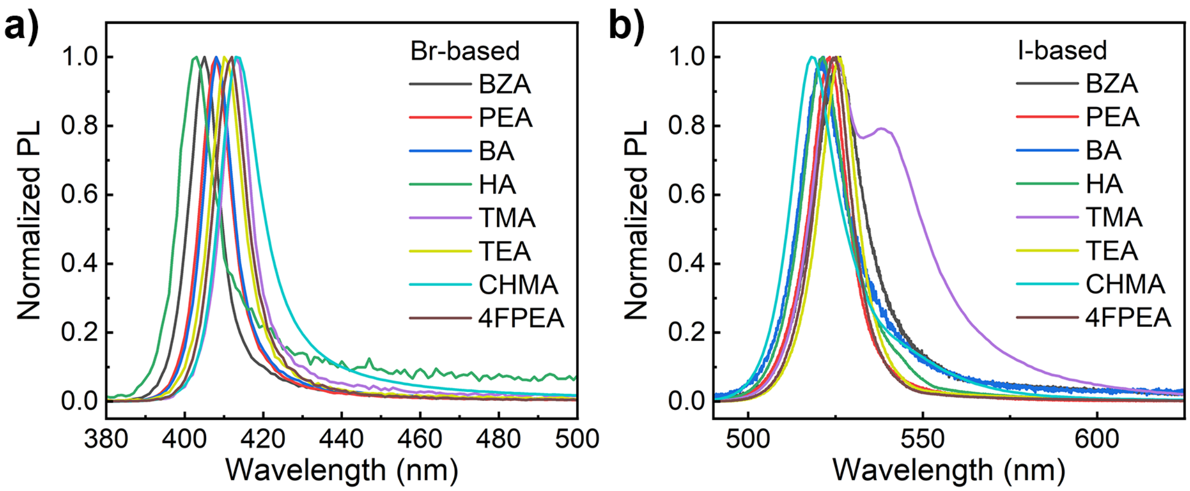

Figure S1. Normalized PL spectra of a) A_2PbBr_4 and b) A_2PbI_4 2D RP perovskites for different cations A.

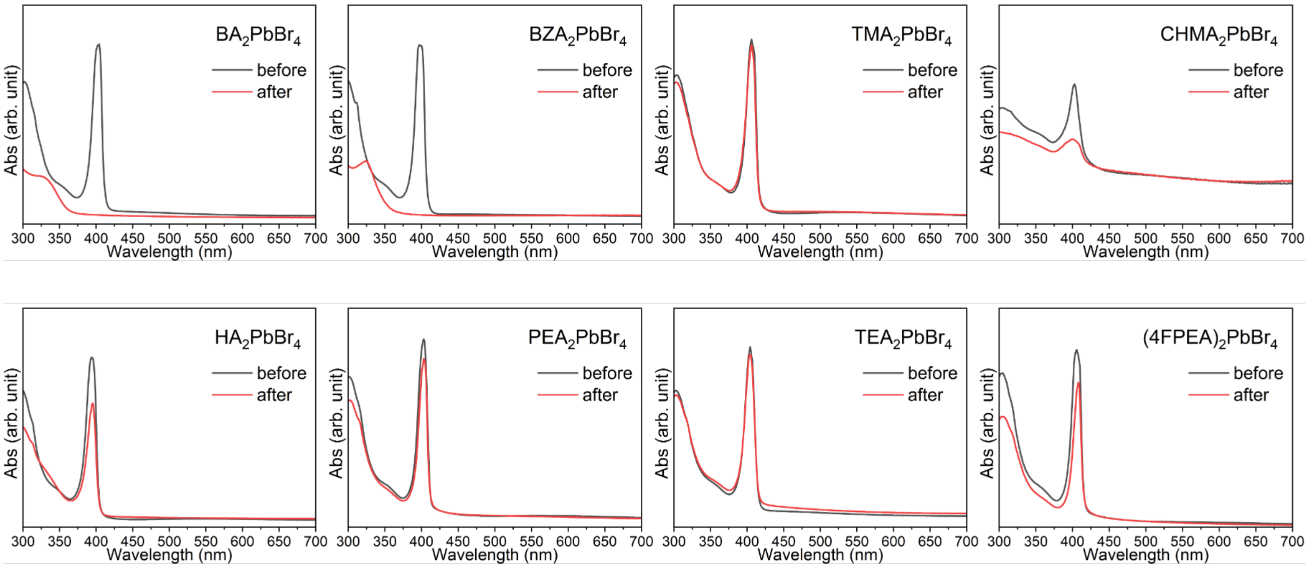

Figure S2. Absorption spectra of A_2PbBr_4 perovskites before and after 120 min 1 Sun illumination in ambient (~60% RH) for different cations A.

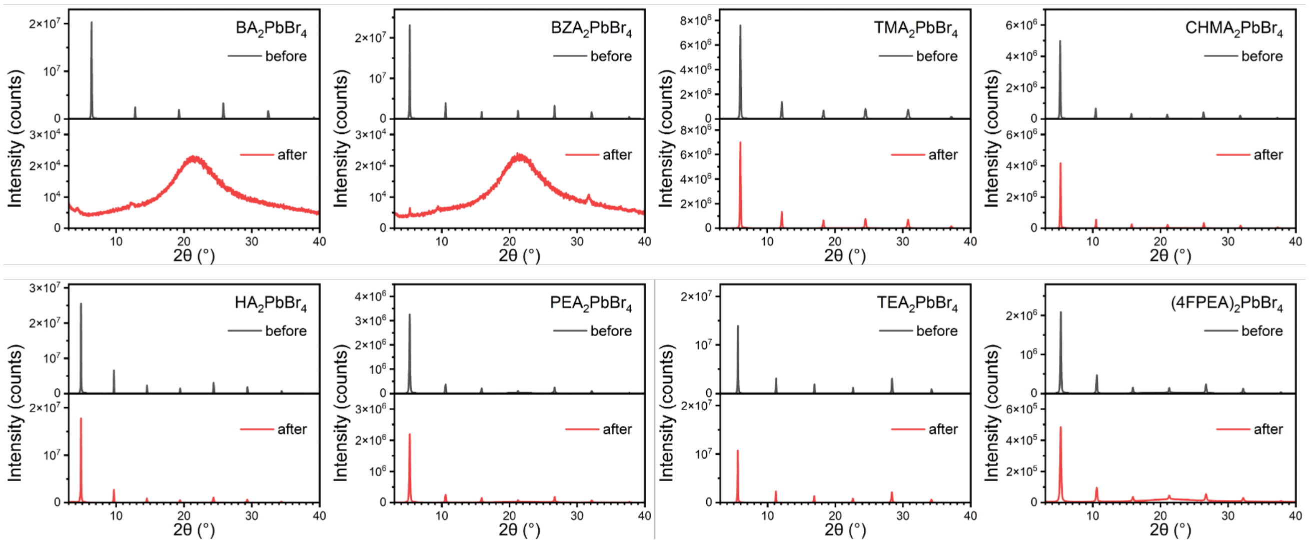

Figure S3. XRD patterns of A_2PbBr_4 perovskites before and after 120 min 1 Sun illumination in ambient (~60% RH) for different cations A.

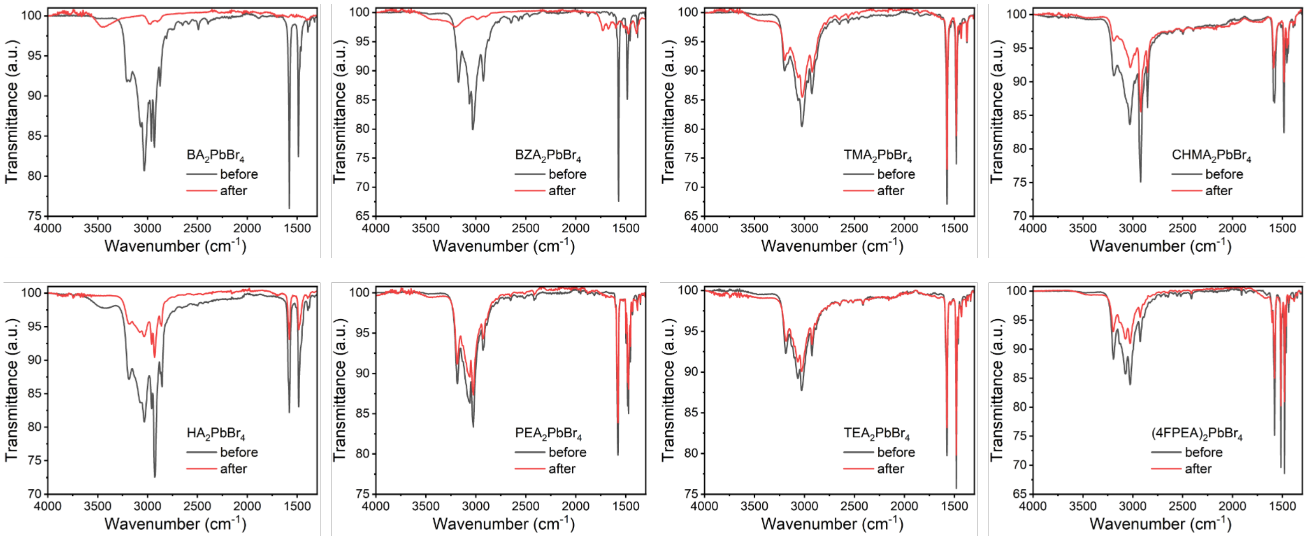

Figure S4. FTIR spectra of A_2PbBr_4 perovskites before and after 120 min 1 Sun illumination in ambient (~60% RH) for different cations A.

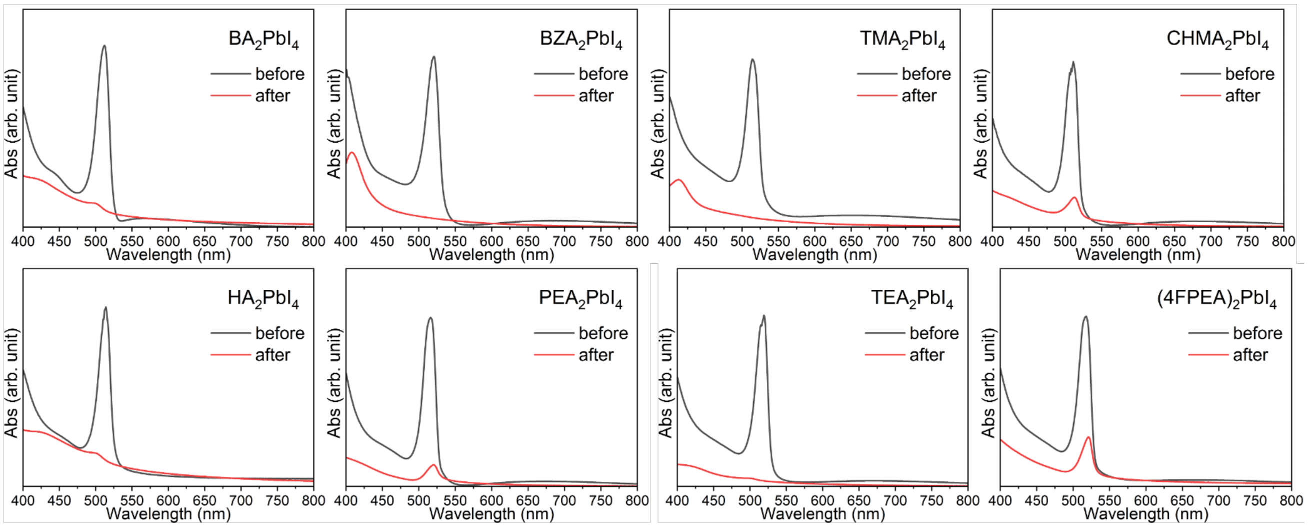

Figure S5. Absorption spectra of A_2PbI_4 perovskites before and after 120 min 1 Sun illumination in ambient (~60% RH) for different cations A.

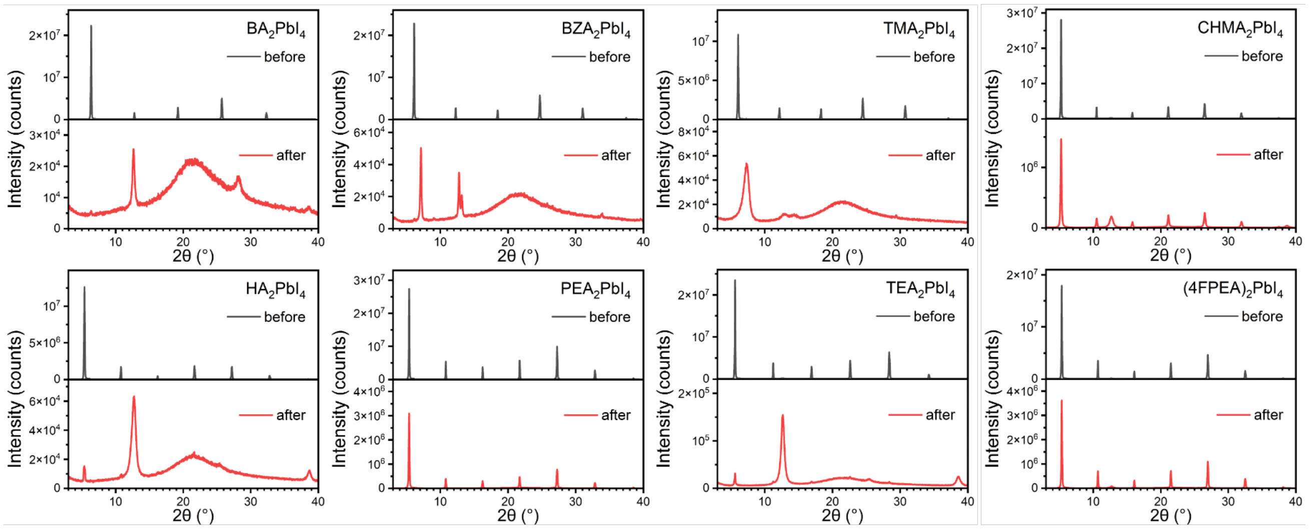

Figure S6. XRD patterns of A_2PbI_4 perovskites before and after 120 min 1 Sun illumination in ambient (~60% RH) for different cations A.

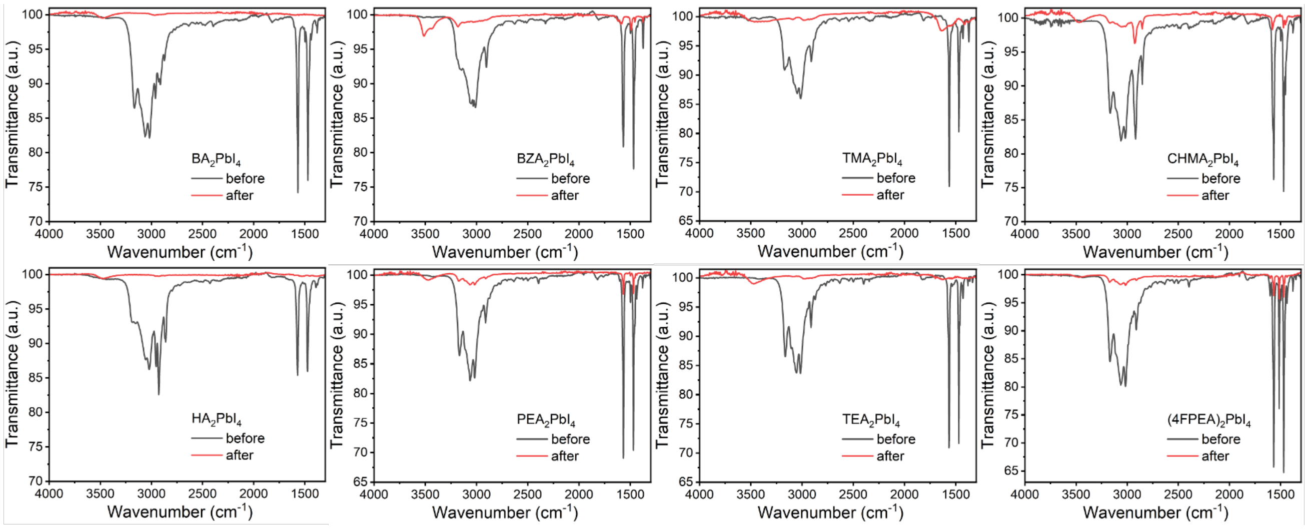

Figure S7. FTIR spectra of A_2PbI_4 perovskites before and after 120 min 1 Sun illumination in ambient (~60% RH) for different cations A.

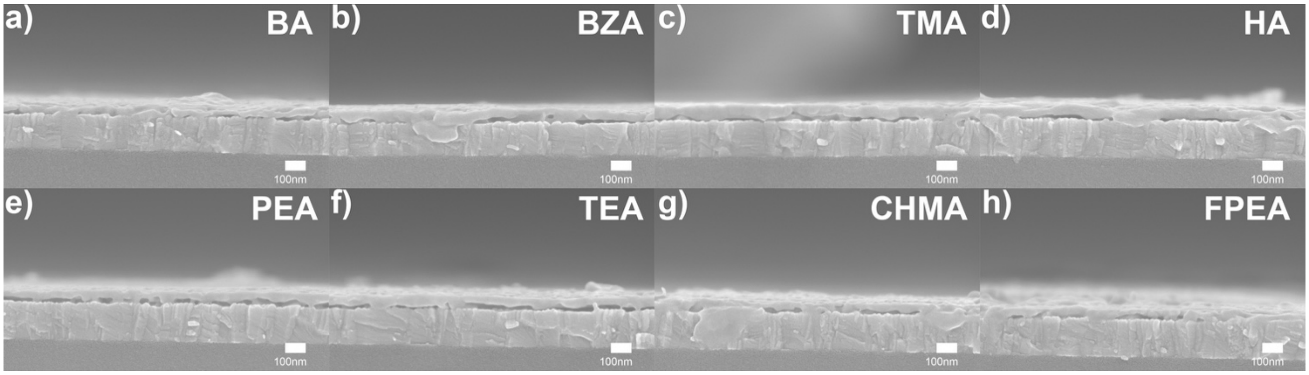

Figure S8. SEM images of cross section of A_2PbBr_4 perovskite films on ITO glass substrates.

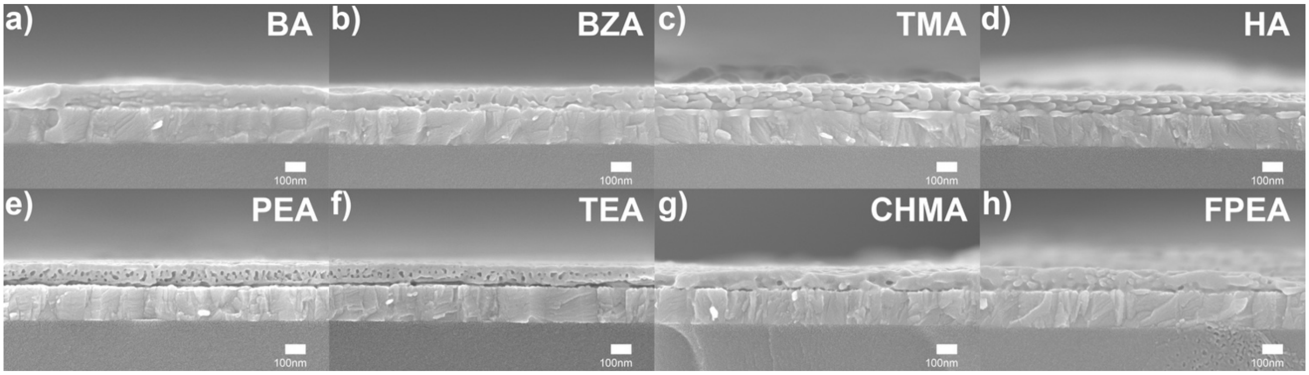

Figure S9. SEM images of cross section of A_2PbI_4 perovskite films on ITO glass substrates.

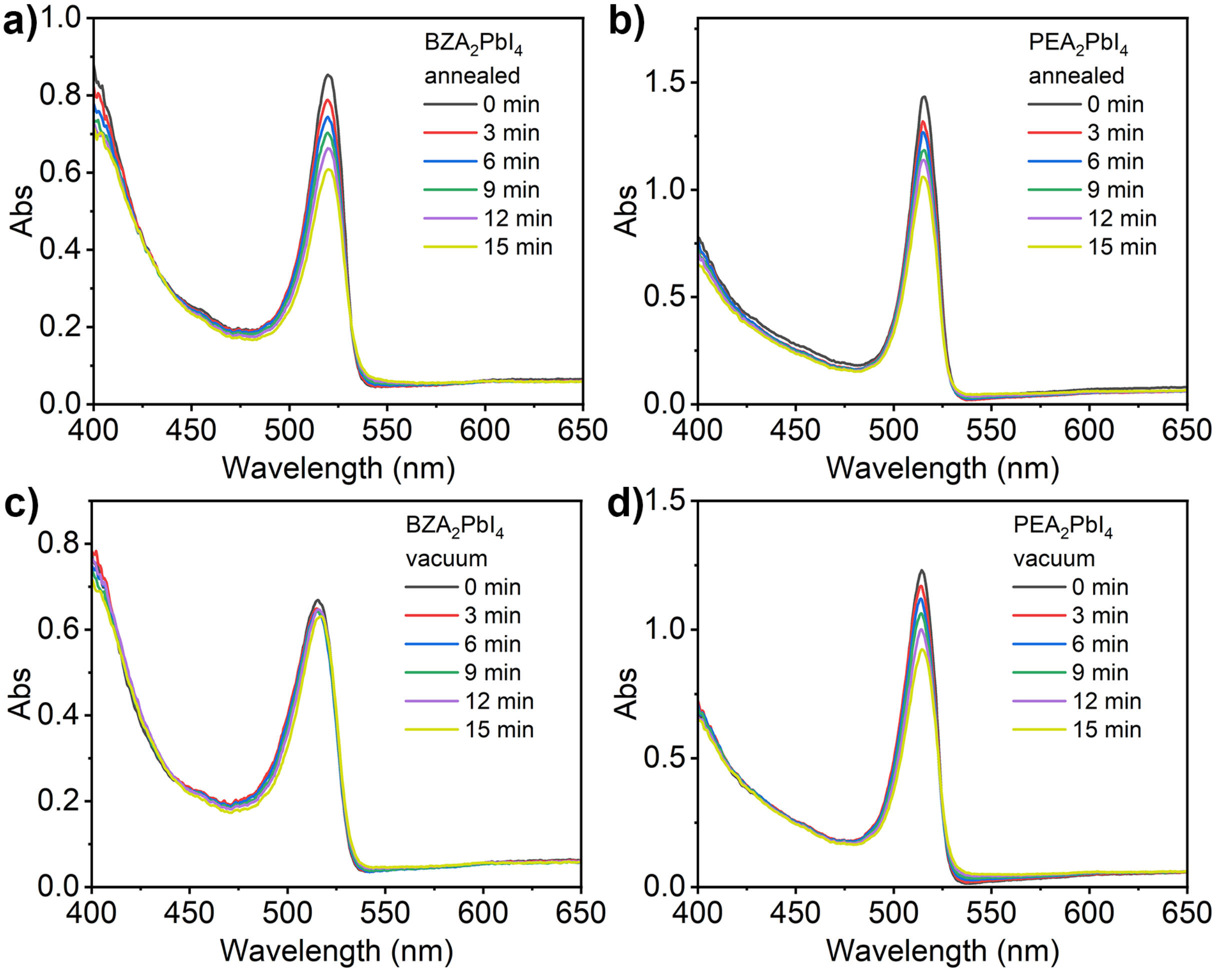

Figure S10. Absorption spectra of a) annealed BZA_2PbI_4 b) annealed PEA_2PbI_4 c) vacuum BZA_2PbI_4 d) vacuum PEA_2PbI_4 for different illumination times in ambient air.

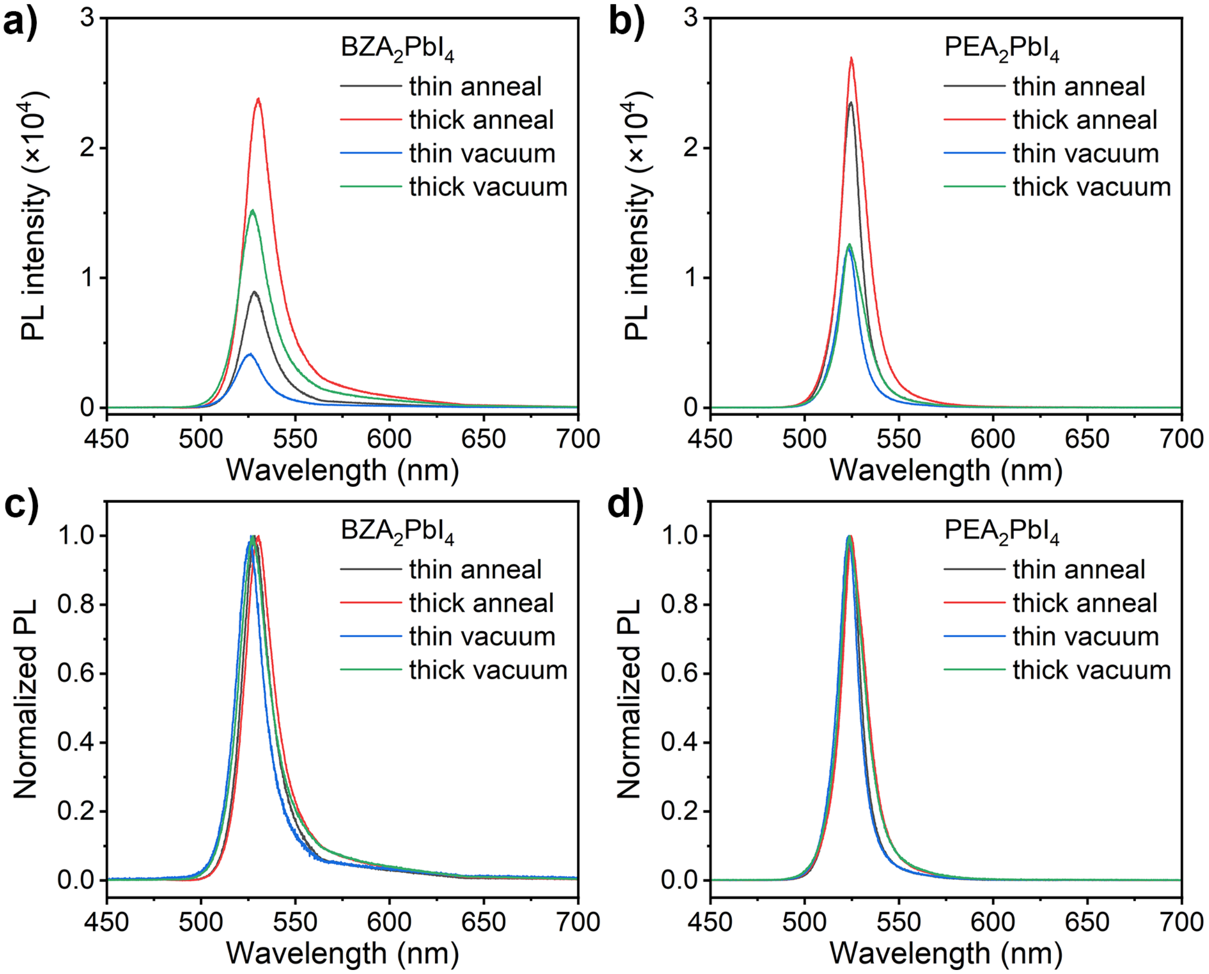

Figure S11. PL spectra of perovskite film with different thickness (thin denotes films prepared from 0.2M solution, and thick denotes films prepared from 0.4M solution) and treatment for a) BZA_2PbI_4 b) PEA_2PbI_4; Normalized PL spectra of perovskite film with different thickness and treatment for a) BZA_2PbI_4 b) PEA_2PbI_4.

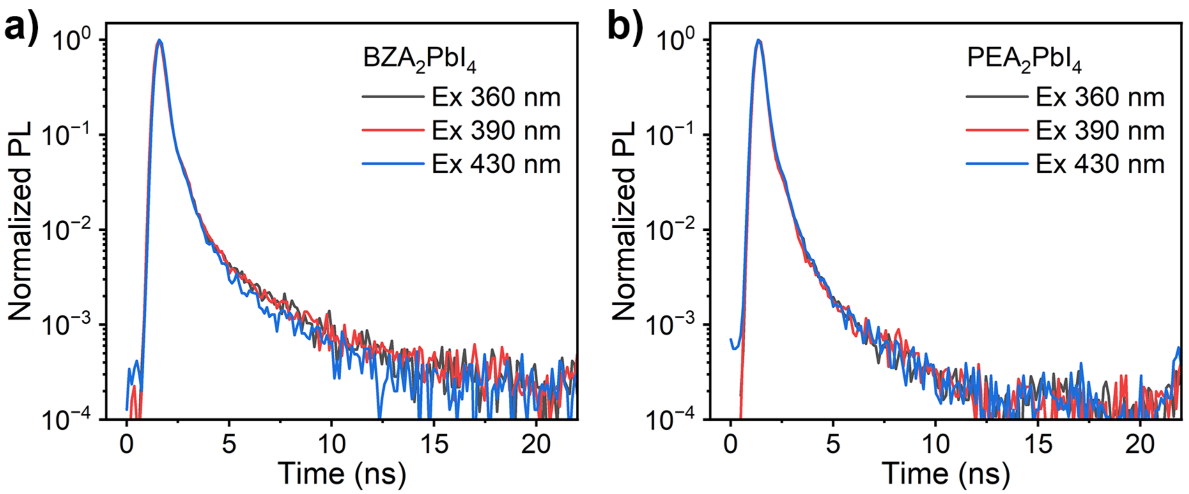

Figure S12. TRPL traces measured for different excitation wavelengths for a) BZA_2PbI_4 b) PEA_2PbI_4.

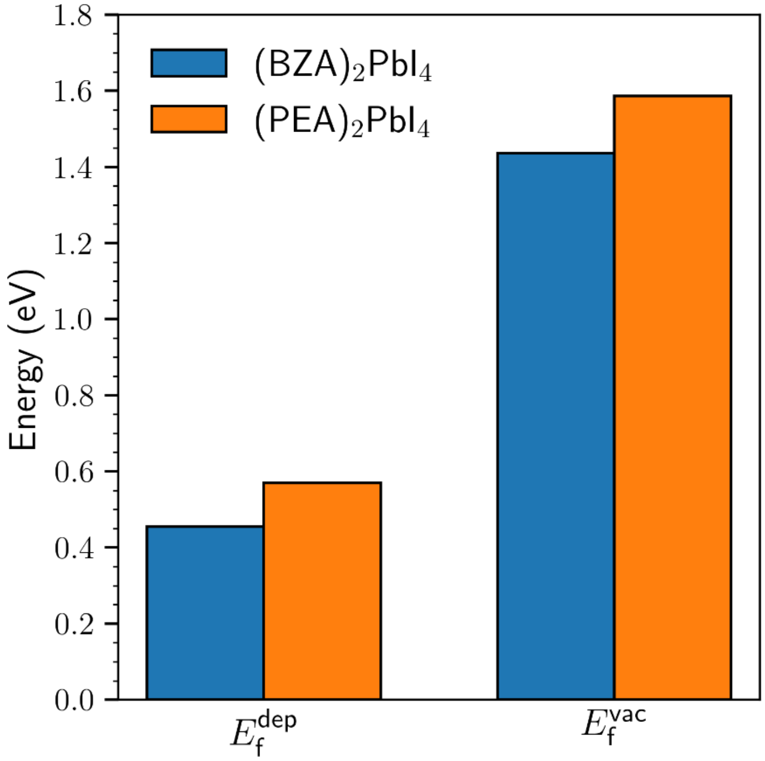

Figure S13. Estimated lower bounds on the deprotonation and organic spacer vacancy formation energies for BZA_2PbI_4 and PEA_2PbI_4.

Table S1. Thickness of different A_2PbBr_4 and A_2PbI_4 films prepared from 0.2M solutions estimated from cross section SEM images shown in **Figures S8** and **S9**. The average values and standard deviations were calculated from measurements at six points.

Perovskites (A_2PbBr_4)	Thickness (nm)	Perovskites (A_2PbI_4)	Thickness (nm)
BA	46.4±7.5	BA	104.8±13.7
BZA	49.7±8.7	BZA	110.4±8.7
TMA	52.3±9.0	TMA	114.4±22.4
HA	56.7±4.0	HA	102.0±12.0
PEA	50.8±4.0	PEA	103.2±11.0
TEA	50.7±6.5	TEA	100.9±10.4
CHMA	52.8±7.6	CHMA	99.0±12.3
FPEA	50.2±6.7	FPEA	98.7±8.0

Table S2. Quantitative characteristics of light-emitting properties PEA_2PbI_4 and BZA_2PbI_4 samples under exposure to laser radiation with different wavelengths and fluences

Sample	PL_{max} (nm)	PL_{FWHM} (nm)	<τ> (ns)	ΔI[a]	k_q (s^{-1})[b]
PEA_2PbI_4 before exposure	517.1±0.05	14.8±0.05	0.362±0.005	-	-
**Exposure at 360 nm**					
PEA_2PbI_4 after exposure @1 µW	516.2±0.05	15.32±0.05	0.35±0.007	0.65±0.01	3.4×10^{-4}
PEA_2PbI_4 after exposure @10 µW	515.1±0.05	14.94±0.05	0.419±0.005	0.17±0.02	1.4×10^{-3}
PEA_2PbI_4 after exposure @75 µW	514.5±0.05	14±0.05	0.401±0.01	0.94±0.03	1.0×10^{-4}
**Exposure at 390 nm**					
PEA_2PbI_4 after exposure @1 µW	515.75±0.05	14.5±0.05	0.313±0.004	0.94±0.03	2.1×10^{-5}
PEA_2PbI_4 after exposure @10 µW	514.9±0.05	14.5±0.05	0.33±0.009	0.42±0.01	6.3×10^{-4}
PEA_2PbI_4 after exposure @75 µW	514.9±0.05	14.5±0.05	0.403±0.009	0.87±0.05	1.7×10^{-4}
**Exposure at 430 nm**					
PEA_2PbI_4 after exposure @1 µW	516.6±0.05	14±0.05	0.33±0.006	0.92±0.03	1.8×10^{-5}
PEA_2PbI_4 after exposure @10 µW	515.3±0.05	14.8±0.05	0.339±0.008	0.7±0.08	2.0×10^{-4}
PEA_2PbI_4 after exposure @75 µW	513.6±0.05	14.8±0.05	0.463±0.011	0.43±0.09	8.0×10^{-4}
BZA_2PbI_4 before exposure	520.8±0.05	17.45±0.05	0.39±0.005	-	-
**Exposure at 360 nm**					
BZA_2PbI_4 after exposure @ 1 µW	520.5±0.1	16.14±0.15	0.34±0.002	0.19±0.002	9.1×10^{-4}
BZA_2PbI_4 after exposure @ 10 µW	520.8±0.5	16.3±0.5	0.34±0.003	0.05±0.005	2.2×10^{-3}
BZA_2PbI_4 after exposure @ 75 µW	520.4±0.3	16.8±0.7	0.34±0.005	0.032±0.005	3.9×10^{-3}
**Exposure at 390 nm**					
BZA_2PbI_4 after exposure @ 1 µW	521.1±0.05	20.4±0.05	0.30±0.004	0.51±0.01	3.7×10^{-4}
BZA_2PbI_4 after exposure @ 10 µW	522.2±0.1	20.9±0.25	0.31±0.003	0.16±0.005	9.7×10^{-4}
BZA_2PbI_4 after exposure @ 75 µW	521.8±0.3	18.3±0.5	0.32±0.003	0.042±0.001	1.7×10^{-3}
**Exposure at 430 nm**					
BZA_2PbI_4 after exposure @ 1 µW	521.5±0.05	18.3±0.05	0.30±0.007	0.66±0.02	2.4×10^{-4}
BZA_2PbI_4 after exposure @ 10 µW	521.2±0.05	18.6±0.07	0.29±0.005	0.35±0.01	5.7×10^{-4}
BZA_2PbI_4 after exposure @ 75 µW	521±0.2	19±0.45	0.30±0.005	0.072±0.005	1.3×10^{-3}

[a]the ratio of PL intensities after and before exposure

[b]amplitude-averaged quenching rate estimated from multiexponential fit of kinetic curves provided in **Figure 5** and **Figure 6**.
